# Mechanism of Immunoregulatory Properties of Vasoactive Intestinal Peptide in the K/BxN Mice Model of Autoimmune Arthritis

**DOI:** 10.3389/fimmu.2021.701862

**Published:** 2021-07-16

**Authors:** Javier Leceta, Marina I. Garin, Carmen Conde

**Affiliations:** ^1^ Department of Cell Biology, Faculty of Biology, Complutense University of Madrid, Madrid, Spain; ^2^ Division of Hematopoietic Innovative Therapies, Centro de Investigaciones Energéticas, Medioambientales y Tecnológicas (CIEMAT) and Centro de Investigación Biomédica en Red de Enfermedades Raras (CIBER-ER), Madrid, Spain; ^3^ Advanced Therapy Unit, Instituto de Investigación Sanitaria Fundación Jiménez Díaz (IIS-FJD/UAM), Madrid, Spain; ^4^ Laboratorio de Reumatología Experimental y Observacional, Instituto de Investigación Sanitaria de Santiago (IDIS), Hospital Clínico Universitario de Santiago de Compostela (CHUS), SERGAS, Santiago de Compostela, Spain

**Keywords:** neuroimmunology, VIP, T cell plasticity, follicular regulatory T cells (Tfr), nonclassic Th1 cells

## Abstract

The K/BxN mouse model of rheumatoid arthritis (RA) closely resembles the human disease. In this model, arthritis results from activation of autoreactive KRN T cells recognizing the glycolytic enzyme glucose-6-phosphate isomerase (GPI) autoantigen, which provides help to GPI-specific B cells, resulting in the production of pathogenic anti-GPI antibodies that ultimately leads to arthritis symptoms from 4 weeks of age. Vasoactive intestinal peptide (VIP) is a neuropeptide broadly distributed in the central and peripheral nervous system that is also expressed in lymphocytes and other immune cell types. VIP is a modulator of innate and adaptive immunity, showing anti-inflammatory and immunoregulatory properties. Basically, this neuropeptide promotes a shift in the Th1/Th2 balance and enhances dedifferentiation of T regulatory cells (Treg). It has demonstrated its therapeutic effects on the collagen-induced arthritis (CIA) mouse model of RA. In the present hypothesis and theory article, we propose that the immunoregulatory properties of VIP may be due likely to the inhibition of T cell plasticity toward non-classic Th1 cells and an enhanced follicular regulatory T cells (Tfr) activity. The consequences of these regulatory properties are the reduction of systemic pathogenic antibody titers.

## Introduction

The lymphoid tissue associated with the intestine constitutes the largest accumulation of cells of both the innate immune system and the adaptive immune system of the body. Local cytokine production forms an environment that influences the differentiation of distinct T cell subsets, conditioning local and systemic immune responses. Notably, the development of T cell subsets, especially Th17 and Treg cells, is broadly influenced by commensal bacterial species (1–3). Innate immune cells in these locations sense environmental cues, produce cytokines, and interact with T cells, directing the differentiation of the various T cell subsets (4, 5). The migration of these latter cells determines the type of immune response both locally and systemically.

The gastrointestinal tract is highly innervated by the parasympathetic and sympathetic systems (6). Also, the autonomous enteric nervous system constitutes an extensive neuronal network (7). All these nerve terminals are in proximity with the lymphoid tissue at this location. Immune cells express receptors for nervous mediators, indicating an integrated neuro-immune communication of particular significance in the intestine. Neurotransmitters, such as norepinephrine or serotonin, and neuropeptides, such as SP, VIP, CGRP, or neuromedin, are found in the nervous system associated to the intestine (8). It has been shown that these inputs balances type 1, type2, and type 3 immune responses, regulating multi-organ homeostasis (9).

The aim of this hypothesis and theory article is to settle the effect of VIP in the humoral immune response and the Th17 to Th1 plasticity. Also, we propose its role in the enhancement of Tfr cell activity.

## The K/BxN Mice Model of Rheumatoid Arthritis

RA is an autoimmune inflammatory disease that results in chronic inflammation and tissue damage in the joints. Its dependence on T cells has been demonstrated in several animal models, and Th1, as well as Th17 cells, has been implicated in the etiology of the disease. The role of humoral immunity in the pathogenesis of arthritis has also been underlined; the generation of autoantibodies against citrullinated proteins (ACPA) is a landmark of RA (10). In animal models, autoantibodies are efficient by themselves to induce the disease. Autoantibodies transferred can induce RA, suggesting that T or B lymphocyte responses are required for the induction of RA. In this way, an induction phase, dependent on adaptive immunity, and an effector phase, mediated by antibodies and innate immunity, can be delineated. An imbalance between different Th subsets has been implicated, triggering the pathology.

The K/BxN mouse model of spontaneous arthritis shares immunological abnormalities with human RA. K/BxN mice proceed from a TCR transgenic mouse (KRN-C57BL/6) crossed with NOD mice. The KRN TCR in the NOD-derived Ag7 MHC class II molecules recognizes the ubiquitously expressed protein glucose-phosphate isomerase (GPI) (10–13). K/BxN mice develop severe arthritis with a rapid onset at 4 to 5 weeks. B cell function is also crucial in this animal model because autoantibodies against GPI present in the serum, mainly of the IgG1 isotype, transfer the disease (14, 15). The contribution of T-cell subsets to this pathology has been extensively studied. Although RA was originally attributed to increased Th1 cells, it was shown that Th2 cells and their IL-4 production were necessary to develop arthritis in this model (16). With the discovery of Th17 cells, this cell type has been shown to participate in the pathogenesis of RA. In the K/BxN model, Th17 cell development has been shown to be dependent on gut microbiota, and it is necessary to elicit high anti-GPI antibody titers (17, 18). In germ-free condition, K/BxN mice have decreased Th1 and Th17 subpopulations. Other authors, however, diminish the participation of Th17 cells in the pathology and describe Tfh cell differentiation, mediating autoantibody production and arthritis development. Tfh cells are also regulated by microbiota, and using this animal model shows that Peyer’s patches Tfh cells were essential to induce systemic anti-GPI antibody titers of the IgG1 isotype in response to commensal segmented filamentous bacteria (19, 20).

In any case, the K/BxN mice is the ideal model to study the interplay between the immune system and their different cell population and gut microbiota, as well as other local immunoregulatory system, such as the nervous system, which are closely tied at these locations. The balance among all these factors has been proposed to determine the etiology of different inflammatory and autoimmune diseases.

## Immunoregulatory Properties of VIP

Vasoactive intestinal peptide is a 28-amino acid neuropeptide initially isolated from the intestine, which has vasodilator properties (21). It is widely distributed in neurons of the central and peripheral nervous systems, especially in the gastrointestinal tract (22). It belongs to the secretin/glucagon family and binds with high affinity to two receptors, VIPR1 and VIPR2 (23). While most cells of the immune system express VIPR1 receptors constitutively, VIPR2 is induced upon activation (24). The immunoregulatory properties of VIP have been investigated for more than 20 years. It has been determined that VIP inhibits the production of the inflammatory cytokines TNFα, IL-6, IL-12, or chemokines produced by immune cells. Other cytokines, such as IL-10, TGFβ, or IL-1Ra, are induced in the presence of VIP (25). It also modifies the polarity of Th responses, favoring Th2 and inhibiting Th1, as evidenced by the levels of IL-4 and IFNγ produced during the immune response (26, 27). Its efficacy in the treatment of several models of inflammatory and autoimmune responses, such as rheumatoid arthritis, multiple sclerosis, inflammatory bowel disease, and type 1 diabetes, has been widely demonstrated (28–32).

In this sense, the therapeutic benefit of this neuropeptide was attributed to a shift in the Th1/Th2 balance and the enhancement of differentiation of T regulatory cells (Treg) (25, 32). With the discovery of the implication of Th17 cells in these pathologies, the re-evaluation of these aspects indicated that VIP reduces the pathogenic profile of the Th17 cells, decreasing their Th1 potential, an effect accompanied by an increase in the Treg/Th17 balance in human lymphocytes obtained from early arthritis patients (33–35). The recent finding of the plasticity among different T cell types previously identified begs the re-evaluation of the different cell types implicated in these pathologies and their clinical implications. Especially relevant is the plasticity of Th17 cell that may shift to a Th1-like or a Treg phenotype. Also, the stable suppressor phenotype of Treg cells is conditioned by cytokines in the tissue microenvironments (36, 37). On the other hand, the effect of VIP on the humoral immune response and the B cells compartment has been scarcely studied, and this deficiency must be corrected.

## Hipothesis: VIP Inhibits The Plasticity of Th17 Cell Toward the Non-Classic Th1 Cells and Potentiates T Follicular Regulatory Cells

Our knowledge about the modulation of the different T cell subsets by VIP paralleled the discovery and knowledge of the different lymphoid cell subpopulations. In the era of the Th1/Th2 paradigm, the first studies on the immunoregulatory role of VIP, dated in 2001, was attributed to its effect on the balance between these two populations in different animal models, increasing the magnitude of Th2 responses and decreasing the magnitude of Th1 responses (29). In 2005, Th17 cells were reported as a novel Th cell playing an important role in the pathogenesis of autoimmune diseases, including RA. The effect of VIP on Th17 cells has been controversial. In the CIA model VIP downregulated Th17 responses (38, 39). In addition to these observations, other studies indicated that this neuropeptide induced Th17 differentiation (40). Afterward, Th17 cells were shown to pose a high degree of phenotypical and functional plasticity, depending on the cytokine microenvironment. Under inflammatory conditions, the Th17 profile is unstable and can shift to Th17/Th1 or Th1 phenotype in human arthritis. Later, Th17/Th1 cells were shown to have a pathogenic role (41, 42). More recent studies have indicated that VIP maintained the non-pathogenic profile of human Th17 polarized cells, decreasing their Th1 potential (34, 35). Th17 cells may lose their markers becoming phenotypical Th1-like cells. These ex-Th17 cells are now named as non-classical Th1 cells.

Another aspect of the Th17 plasticity is the transdifferentiation between this cell subset and Treg cells. TGFβ is required for their differentiation, and their master transcription factors are transiently co-expressed early during their differentiation. Treg cells can be broadly classified into two groups (43): natural Treg cells (nTreg) generated in the thymus that show T cell receptors with high affinity for self-antigens, and peripherally induced Treg cells (iTreg) developed from conventional naïve CD4+ T cells in the periphery after antigen encounter in the presence of specific factors (44). Multiple studies have shown that VIP can induce the generation of Treg cells. In this sense, some authors have demonstrated the expansion of nTreg by VIP, whereas other studies have suggested that VIP is implicated in the generation of iTreg cells (45–48).

Considering previous data and new experimental evidence, the effect of VIP on the different T cell population must be re-evaluated including the abovementioned proposed hypothesis. To find support for the abovementioned hypothesis, we took advantage of a running experiment on the effect of VIP in the humoral immune response in the K/BxN model.

## Experimental Evidence

**Figure 1:**
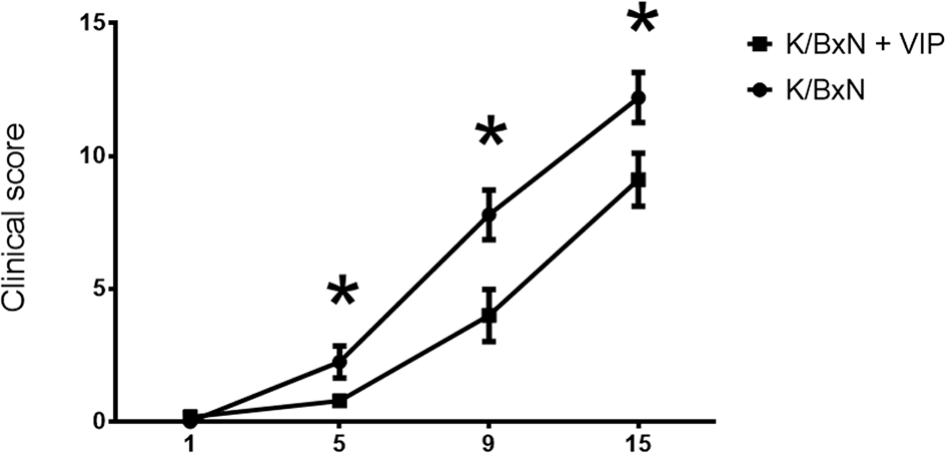
Time course of clinical score values (mean ± SEM) in untreated and VIP treated K/BxN mice. X axis represents days of treatment (beginning on day 21 of age). *p < 0.05.

Autoantibodies are the hallmark of RA, and ACPA antibodies are highly specific in humans during the progression of the disease. The development of arthritis in the K/BxN mouse model is dependent on antibodies directed against the ubiquitously expressed protein GPI (49). Arthritis in K/BxN mice is dependent on both, innate and adaptive immunities. It can be divided in two phases: inductive and effector phases. In the inductive phase, autoreactive T and B cells are activated, resulting in the production of autoantibodies in the effector phase. Transfer of serum from arthritic K/BxN mice can induce the development of arthritis in any mouse strains. Immune complexes trigger complement activation and the recruitment of innate cells to the joint (12, 13). Shortly after weaning, arthritogenic T cells appear in the spleen between 3 and 4 weeks of age and arthritis onset can appear by 4 weeks of age. The effector phase, on the other hand, depends on autoantibody titers against GPI. Because VIP has been shown to be effective in preventing arthritis in the CIA (29), we wanted to know if VIP also has a beneficial effect on antibody titers against the autoantigen GPI. So, we treated K/BxN mice 5 days a week for 2 weeks i. p. with 2 nM of VIP from 21 days of age, an age at which anti-GPI antibodies have began to appear (15). A similar VIP dose can delay insulitis and prevent development of diabetes in NOD mice administered after weening (32). Our results indicate that VIP-treated K/BxN mice showed a milder arthritis, with significantly lower clinical score than the untreated mice (**Figure 1**).

**Figure 2:**
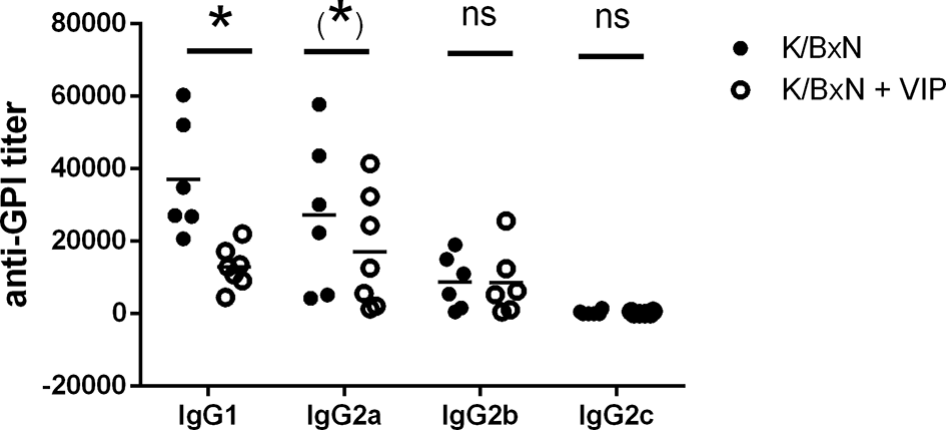
Anti-GPI titers of different isotypes in K/BxN mice treated with PBS or VIP for 15 days. Mean is shown as the horizontal line within each group; each symbol represents an individual mouse. *p < 0.05; *0.05 < p < 0.1; ns, not significant.

Therefore, we looked for total anti-GPI by ELISA. The humoral immune response is dominated by IgG antibody titers, as previously reported, with very low titers of IgM antibodies. IgE or IgA antibodies were undetectable. Serum anti-GPI IgG antibody titer in VIP-treated K/BxN mice decreased by one order of magnitude compared with untreated K/BxN mice. Of the different anti-GPI Ig isotypes, only IgG has been associated with the arthritogenic pathology, and different isotypes of IgG participate differently on it, with IgG1 being arthritogenic. To determine the influence of VIP, we examined serum anti-GPI IgG levels of the different isotypes (**Figure 2**). The dominant IgG isotypes were IgG1 and IgG2a, with lower levels of IgG2b, and very low levels of IgG3 in untreated mice, as previously stated. VIP decreases significantly the IgG1 anti-GPI antibody titers and also that of IgG2a, which is close to reaching statistical significance. IgG2b and IgG2c were not affected by VIP.

**Figure 3:**
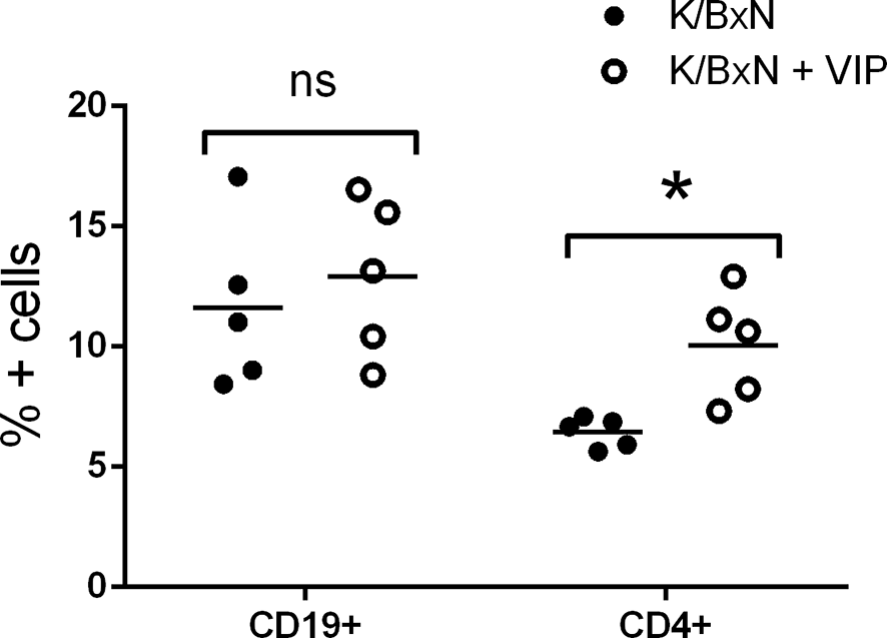
Flow cytometry analysis of spleen lymphoid cells of K/BxN mice treated with PBS or VIP. Mean is shown as the horizontal line within each group; each symbol represents an individual mouse. *p < 0.05.

Although the effector phase of arthritis is triggered by pathogenic autoantibodies, the induction phase is mediated by different subsets of Th cells that provide help for the different Ig isotypes. Th2-Tfh cells are implicated in the production of IgG1, whereas IgG2a and IgG2b secretion is mediated by Th1 and Th17 cells, respectively (47, 48). In K/BxN mice, autoreactive KRN T cells escape negative selection in the thymus and are activated in the periphery by GPI where they provide help to GPI-reactive B cells (14, 50). So, T cells are required for arthritis development, especially at the inductive phase. B cells produce arthritogenic autoantibodies that are necessary and sufficient for arthritis development during the effector phase. We surveyed how VIP treatment affected T and B cell populations, analyzing both cell subpopulations in the spleen because GPI antibody-secreting cells reside mostly in this organ (17). Flow cytometry analysis demonstrated that the frequency of B cells were similar in VIP-treated and untreated K/BxN mice. However, the percentages of T CD4+ cells were higher in VIP-treated K/BxN mice than in the non-treated K/BxN mice (**Figure 3**).

**Figure 4:**
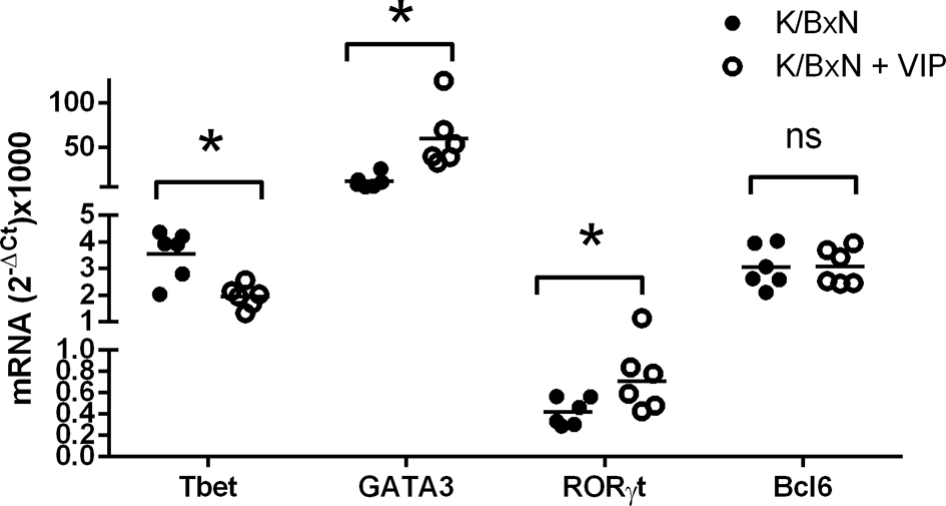
mRNA expression of master transcription factors of different Th cell subsets of PBS or VIP treated K/BxN mice. The expression was determined by quantitative real-time PCR as indicated (supplemental materials). Mean is shown as the horizontal line within each group; each symbol represents an individual mouse. *p < 0.05; ns, not significant.

These data suggest that VIP targets adaptive T responses during the induction phase of the disease. For antibody response, different Th cell subsets influence the isotype of the antibody response mounted by B cells. Thus, as Th2-Tfh cells are implicated in the production of IgG1, and Th1 and Th17 mediate the production of IgG2a and IgG2b, we have studied the gene expression of the master transcription factors and cytokine signature of the Th types by RT-PCR in the spleen to assess the participation of the different Th subpopulations in the immune response of K/BxN mice and the effect of VIP. **Figure 4** shows that the immune response in untreated K/BxN mice were dominated by the Th2 cell subset, according to the gene expression of GATA3, but Th1 and Tfh responses are also highly expressed, that is in accordance with high anti-GPI IgG1 and IgG2a serum levels. However, the Th17 master transcription factor Rorγt is expressed at a much lower levels, in parallel to the lower anti-GPI IgG2b and IgG2c serum levels. Treatment of K/BxN mice with 2 nM of VIP 5 days a week for 2 weeks from the very early phase of arthritis development has no effect on splenic Tfh marker Bcl6 but significantly reduced the expression of the Th1 master transcription factor Tbet, (**Figure 4**). The Th2 master transcription factor GATA3, however, is significantly increased. Also, the Th17 master transcription factor Rorγt was significantly increased, resulting in a Th17/Th1 balance more skewed toward Th17 function.

Two types of Th1 cells have been described: bona fide Th1 cells and non-classic Th1 (ex-Th17) cells, the latter derived from Th17 cells (51). The pathogenic activity of Th17 cells has been shown to be mediated by their conversion *in vivo* into Th1 cells in animal models of autoimmunity (12, 36). It has been also reported that Th17 cells shift to a Th1 phenotype under inflammatory environments in rheumatoid arthritis (42). Non-classical Th1 cells are not constrained by Treg cells (49). We hypothesize that the effect of VIP on Th1 cells reported in this study may be mediated by the inhibition of the transdiferentiation of Th17 cells into Th1 cells, resulting in the reported increase of Th17 cells as indicated by the rise in the expression of Rorγt and a decrease in the expression of the Th1 master transcription factor Tbet, as well as lower levels of IgG2a antibodies. The higher accumulation of Th17 cells and a decrease in Th1 cells in VIP treated mice may indicate the decreased plasticity between both cell subsets. Our hypothesis is supported by the reported effect of VIP inducing the differentiation of Th17 cells (40). We have also shown previously that VIP increases the differentiation human Th17 cells and inhibit their bias toward Th1-like cells (38).

Comparing the expression of T cell subpopulation markers with the antibody titers of the different isotypes, we may conclude that lower antibody titers of IgG2a isotype may be explained by the reduction of the Th1 marker Tbet. However, the great reduction of the IgG1 isotype does not match the increased expression of the Th2 marker GATA3. So, we considered the possibility of alterations in the population of Bcl6 Th cells. The Bcl6 transcription factor is expressed by both Tfh and Tfr cells. Tfh cells are characterized by the expression of the master transcription factor Bcl6 and provide germinal center B cells with signals that culminate in class switch recombination and differentiation of plasma cells that produce large quantities of isotype class switch of high affinity IgG1 antibodies. Tfr cells regulate the GC reactivity (52) and suppress the effects of Tfh cells on antibody response, without affecting the expression of the master transcription factor Bcl6 in a contact-dependent manner (53).

**Figure 5:**
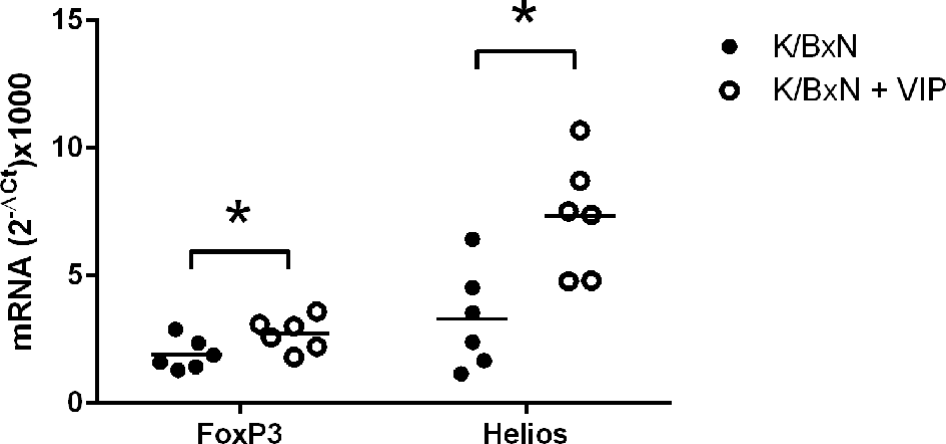
Effect of VIP on mRNA expression of master transcription factors of Treg cells. The expression was determined by quantitative real-time PCR as indicated (supplemental materials). Mean is shown as the horizontal line within each group; each symbol represents an individual mouse. *p < 0.05.

Treg cells can be broadly classified into two groups. Natural Treg cells (nTreg) are generated in the thymus and show T-cell receptors with high affinity for self-antigens. Peripherally induced Treg cells (iTreg) developed from naïve CD4+ T cells in the periphery after antigen encounter in the presence of specific factors (43, 51). All Treg cells express the master transcription factor FoxP3, and iTreg cells differentiate in the periphery from Foxp3 negative T cells (54). The expression of the transcription factor Helios was once thought to discriminate natural from peripherally induced Treg cells (55). In any case,Helios regulates Treg functional stability, and targeted mutation of the Ikaros transcription factor family shows T-cell hyperproliferation, autoantibodies, and elevated IgG serum levels (56). Multiple studies have shown that VIP induce the generation of Treg cells, and both nTreg and iTreg cells have been implicated in different experimental models, as well as in human pathologies (45–48). It is likely that Tfr is implicated in the beneficial effects of VIP reported here in the K/BxN arthritis model. Tfr cells are derived from thymic nTreg cells and express both Foxp3 and Helios (57). This cell type suppresses the effects of Tfh cells on antibody response without affecting the expression of the master transcription factor Bcl6 in a contact-dependent manner (58). In this regard, we have studied the expression of the abovementioned transcription factors. We have found that the transcription factors FoxP3 and Helios are fairly expressed in arthritic K/BxN mice. VIP treatment results in a significative increase of the Treg master transcription factors Foxp3 and Helios (**Figure 5**). There is higher expression of the Treg master transcription factors Foxp3 and Helios, no changes in the Tfh master transcription factor Bcl6 and an important decrease in the antibody titers of the IgG1 isotype points to an enhanced Tfr cell activity by VIP treatment.

## Conclusion

IgG1 has been characterized as the antibodies mediating the effector phase of RA in K/BxN mice. Here, we report a drastic reduction of the titers of this isotype, as well as IgG2a antibody titers, after VIP treatment. We hypothesize that this effect is mediated by a decreased plasticity of Th17 cells to non-classical Th1 cells and together with an enhanced Tfr cell response. This is compatible with the decreased Th17 cell population and increased Tfh cell population seen in the K/BxN model occurring during ageing, which also depends on the gut microbiota. Very recently, gut microbiota has been shown to influence systemic Tfr cells, impacting systemic autoimmunity in the present animal model of autoimmune arthritis (59). This means the implication of the local microbiota and nervous circuits in the regulation of autoimmune diseases.

## Data Availability Statement

The raw data supporting the conclusions of this article will be made available by the authors, without undue reservation.

## Ethics Statement

The animal study was reviewed and approved by Animal Care and Use Committee of the University of Santiago de Compostela.

## Author Contributions

JL designed the research, performed the analytical studies, and wrote the manuscript. MG performed the flow cytometry analyses and critically edited the manuscript. CC performed the animal treatment took the samples and critically edited the manuscript. All authors contributed to the article and approved the submitted version.

## Funding

This work was supported by Instituto de Salud Carlos III grant (PI17/01161), and cofunded by the European Regional Development Fund (ERDF) and by Instituto de Salud Carlos III-ISCIII/PI1701660/Cofinanciado FEDER.

## Conflict of Interest

The authors declare that the research was conducted in the absence of any commercial or financial relationships that could be construed as a potential conflict of interest.
