# Supplementary material for: Mechanism of Immunoregulatory Properties of Vasoactive Intestinal Peptide in the K/BxN Mice Model of Autoimmune Arthritis
Source: Front Immunol. 2021 Jul 16;12:701862. doi: 10.3389/fimmu.2021.701862 (PMC8322839; doi:10.3389/fimmu.2021.701862)
Supplement: Supplementary file 1 [file DataSheet_1.docx]

Supplementary Material

**Materials and Methods**

Mice and treatment protocol.

K/BxN mice were generated by crossing B6.KRN TCR transgenic mice to NOD mice. Mice were maintained in the conventional mouse facility of the Medical School of the University of Santiago de Compostela. Animal care was in compliance with Spanish regulations on the protection of animals used for experimental and other scientific purposes (Real Decreto 223/1998). The experimental protocols were approved by the Animal Care and Use Committee of the University of Santiago de Compostela. 3-week-old mice were randomly divided into VIP treated group and vehicle control group and were injected i.p. with 2nM in 0.2ml of PBS or with 0.2ml of PBS respectively for 2 weeks (five days per week). Arthritis was assessed in each of the four limbs every other day by two blinded observers, using a semiquantitative clinical score (0 = no swelling; 1 = slight swelling and erythema of the ankle, wrist or digits; 2 = moderate swelling and erythema; 3 = severe swelling and erythema; and 4 = maximal inflammation with joint rigidity). The maximum possible score was 16 per mouse. After treatment, individuals were euthanized and sera were collected, hind limbs were prepared for histological studies and cell suspensions were prepared from spleens by mechanical methods.

ELISA for anti-GPI total IgG.

Ninety-six–well plates were coated with 5 mg/ml recombinant GPI in PBS overnight at 4°C and blocked with 1% BSA 0.05% Tween-20 in PBS at room temperature. GPI was a generous gift of Dr. Haochu Huang (Department of Medicine and Knapp Center for Lupus and Immunology Research, University of Chicago, Chicago, Illinois, United States of America). After incubating with serial dilutions of serum samples, bound antibodies were detected by incubation with goat anti-mouse specific isotype antibodies (IgM, IgG, IgG1, IgG2a, IgG2b, IgG3, IgM, IgA, and IgE). TMB (3,3', 5,5'-tetrame-thylbenzidine) substrate was used to reveal peroxidase activity. Serum titers were calculated as the serum dilution that gave the calculated EC50 value based on the fitted nonlinear regression for each sample.

Flow cytometry.

Spleen from each mouse was disaggregated mechanically and suspensions of cells were surface stained with FITC-conjugated anti-CD19 and phycoerythrin‐conjugated anti-CD-4 (BD Pharmingen). Cells were incubated with single fluorochrome‐conjugated antibodies or isotype‐matched control antibodies, to compensate for fluorescence emission overlap and nonspecific fluorescence, respectively. Lymphocytes were distinguished by their different forward‐scatter (FSC) versus side‐scatter (SSC) profiles and were electronically gated and scored. Cells were acquired in a BD FACS CantoTM (Becton Dickinson). At least 10,000 events were acquired. Data were analyzed by BD FACSDiva™ (Becton Dickinson) and Flow Jo (FlowJo) softwares.

RNA extraction and quantitative real-time reverse transcriptase PCR.

For total RNA extraction we used TriReagent method (Sigma‐Aldrich), following the manufacturer's recommendations. RNA was resuspended in diethylpyrocarbonate water, quantified with NanoDrop spectrophotometer (NanoDrop products, Wilmington, NC, USA) and stored at −80 °C. Total RNA (2 μg) was reverse transcribed using High-Capacity cDNA Reverse Transcription (Applied Biosystems, Foster City, CA, USA). Quantitative real‐time PCR analysis was carried out using SYBR Green PCR Master Mix (Applied Biosystems) for all genes. β‐Actin was used as the endogenous reference gene. The primer sequences used were as follows: GATA3, 5′‐GGATACCTCTGCACCGTAGCC‐3′ (forward) and GATA3, 5′‐CTGACGGAAGAGGTGGACGT‐3′ (reverse); Tbx21, 5′‐CACTAAGCAAGGACGGCGAA‐3′ (forward) and Tbx21, 5′‐CCACCAAGACCACATCCACA‐3′ (reverse); RORγT, 5′‐GCCTCCTGCCACCTTGAGT‐3′ (forward) and RORγT,5′‐TCTGCCTTCAGCTTTGCCTC‐3′ (reverse); Bcl6, 5′-CCGGCTCAATAATCTCGTGAA-3′ (forward) and 5′-GGTGCATGTAGAGTGGTGAGTGA-3′ (reverse); Foxp3, 5′‐GAAGCTGGGAGCTATGCAGG‐3′ (forward) and Foxp3, 5′‐TGGCTACGATGCAGCAAGAG‐3′ (reverse); Helios, 5'-ACACCTCAGGACCCATTCTG-3' (forward) and 5'-TCCATGCTGACATTCTGGAG-3' (reverse). Amplification was carried out in a 7900 HT Fast Real‐Time PCR Systems apparatus (Applied Biosystems) under the following conditions: 2 min at 50 °C, 10 min at 95 °C, 40 cycles of denaturation at 95 °C for 15 s, and annealing/extension at 60 °C for 1 min. All gene expression levels were normalized using the formula 2-ΔCT.

Statistical analysis.

Data were plotted and analyzed with Prism 6.01 software (GraphPad). Unpaired, twotailed Student t tests were used to determine statistical significance. A p value < 0.05 was considered statistically significant.
